# Novel SARS-CoV-2 Variant in Travelers from Brazil to Japan

**DOI:** 10.3201/eid2704.210138

**Published:** 2021-04

**Authors:** Takahisa Fujino, Hidetoshi Nomoto, Satoshi Kutsuna, Mugen Ujiie, Tetsuya Suzuki, Rubuna Sato, Tsuguto Fujimoto, Makoto Kuroda, Takaji Wakita, Norio Ohmagari

**Affiliations:** National Center for Global Health and Medicine, Tokyo, Japan (T. Fujino, H. Nomoto, S. Kutsuna, M. Ujiie, T. Suzuki, R. Sato, N. Ohmagari);; Tohoku University, Miyagi, Japan (H. Nomoto, T. Suzuki, N. Ohmagari);; National Institute of Infectious Diseases, Tokyo (T. Fujimoto, M. Kuroda, T. Wakita)

**Keywords:** respiratory infections, severe acute respiratory syndrome coronavirus 2, SARS-CoV-2, SARS, COVID-19, coronavirus disease, zoonoses, viruses, coronavirus, Brazil, Japan

## Abstract

Multiple severe acute respiratory syndrome coronavirus 2 (SARS-CoV-2) variants with higher transmission potential have been emerging globally, including SARS-CoV-2 variants from the United Kingdom and South Africa. We report 4 travelers from Brazil to Japan in January 2021 infected with a novel SARS-CoV-2 variant with an additional set of mutations.

Coronavirus disease (COVID-19), caused by severe acute respiratory syndrome coronavirus 2 (SARS-CoV-2) (*1*), has wreaked havoc worldwide. SARS-CoV-2 causes severe respiratory failure, often rapidly in susceptible patients. Moreover, new variants with estimated higher transmission rates have begun circulating globally, such as Variant of Concern 202012/01 (VOC-202012/01) from the United Kingdom and variant 501Y.V2 from South Africa (*2*). The virulence, reinfection potential, antibody response to, and efficacy of vaccines against these strains, are still unknown, posing a risk for future pandemics. We detected a previously unreported SARS-CoV-2 variant strain in a family arriving in Japan from Brazil.

On January 2, 2021, a healthy man in his 40s arrived at Haneda Airport, Tokyo, Japan, from Amazonas state in Brazil via Istanbul, Turkey. At the airport quarantine station, he and the 3 family members traveling with him tested positive for SARS-CoV-2 by quantitative real-time reverse transcription PCR. All 4 were asymptomatic and were accommodated in a government-designated quarantine facility to wait out the required 14-day quarantine. 

On day 2 of their visit, a fever of 37.6°C developed in the man; on day 4, the man had a cough. On day 6, his oxygen saturation (SpO_2_) dropped to 93% on ambient air, and he was transferred to the National Center for Global Health and Medicine, a tertiary care hospital in Tokyo, for respiratory failure. The remaining 3 family members remained asymptomatic and continued to stay at the government-designated accommodation. 

At admission, the patient had a cough and mild malaise. Physical examination was almost normal except for late inspiratory crackles in the bilateral lower lung fields. The patient’s body temperature was 37.4°C; blood pressure was 113/69 mm Hg and pulse rate 108 beats/min. The patient had a regular respiratory rate of 18 breaths/min and an SpO_2_ of 93% on ambient air. Laboratory tests showed a high C-reactive protein level of 10.47 mg/dL (reference range 0.00–0.14 mg/dL), but complete blood counts, renal function, liver function, and coagulation tests all were within reference ranges. Chest radiography and computed tomography showed ground-glass opacities in the lower lobes of both lungs. 

We started the patient on treatment with 200 mg remdesivir, a subcutaneous injection of unfractionated heparin, and 6 mg oral dexamethasone on day 1 of admission. On day 2 of admission, the patient’s fever subsided, and his general condition improved marginally. On day 3, oxygen therapy was not needed, blood tests showed a decrease in C-reactive protein levels, and no adverse side effects of treatment were observed. He continued treatment with 100 mg/d remdesivir and unfractionated heparin until day 5 of admission and dexamethasone until day 7, during which time we observed no flare-up of symptoms.

We subjected the SARS-CoV-2 detected in the case-patient and in his family to whole-genome sequencing. Phylogenetic analysis suggested a novel variant (GISAID [https://www.gisaid.org] reference no. EPI_ISL_792681) belonging to pangolin lineage P.1 with 12 nonsynonymous mutations including K417T, E484K, and N501Y in the receptor-binding domain of the spike protein (N.R. Faria et al., unpub data, https://virological.org/t/genomic-characterisation-of-an-emergent-sars-cov-2-lineage-in-manaus-preliminary-findings/586). In addition, the variant strain we detected in the travelers had the N501Y mutation in the receptor-binding site of the spike protein, as noted in VOC-202012/01 and 501Y.V2, and the E484K mutation, similar to that noted in the 501Y strain.

We did not observe any remarkable difference in the clinical course of this case-patient compared with COVID-19 cases caused by other known SARS-CoV-2 strains. According to multiple modeling analyses, the new VOC-202012/01 variant could be more infectious than previous strains and might have <70% increased transmissibility (*3*–*5*). Moreover, PCR testing and genomic analysis for this strain suggested an increased viral load in VOC-202012/01 variant. Another strain, 501Y.V2 from South Africa, also has been suggested to have increased transmissibility (H. Tegally et al., unpub. data, https://doi.org/10.1101/2020.12.21.20248640). However, to date, no definitive evidence has shown that either VOC-202012/01 or 501Y.V2 are associated with more severe COVID-19 cases. 

The symptoms in this patient were relatively mild, although short-term oxygen administration was necessary. Onset of pneumonia a week after the onset of disease also followed the conventional clinical course. However, because the patient was young and had no underlying conditions, this case cannot be generalized.

In conclusion, we identified a novel variant strain of SARS-CoV-2 in 4 travelers from Brazil. Variant strains are appearing across the world now, and quarantine systems need to be strengthened. We hope to elucidate the infectivity, pathogenicity, and relationship of SARS-CoV-2 variants to vaccines while continuing to take conventional precautions against novel variant strains.
